# A New Highly Sensitive Method to Assess Respiration Rates and Kinetics of Natural Planktonic Communities by Use of the Switchable Trace Oxygen Sensor and Reduced Oxygen Concentrations

**DOI:** 10.1371/journal.pone.0105399

**Published:** 2014-08-15

**Authors:** Laura Tiano, Emilio Garcia-Robledo, Niels Peter Revsbech

**Affiliations:** Section of Microbiology, Department of Bioscience, Aarhus University, Aarhus, Denmark; Scottish Association for Marine Science, United Kingdom

## Abstract

Oxygen respiration rates in pelagic environments are often difficult to quantify as the resolutions of our methods for O_2_ concentration determination are marginal for observing significant decreases during bottle incubations of less than 24 hours. Here we present the assessment of a new highly sensitive method, that combine Switchable Trace Oxygen (STOX) sensors and all-glass bottle incubations, where the O_2_ concentration was artificially lowered. The detection limit of respiration rate by this method is inversely proportional to the O_2_ concentration, down to <2 nmol L^−1^ h^−1^ for water with an initial O_2_ concentration of 500 nmol L^−1^. The method was tested in Danish coastal waters and in oceanic hypoxic waters. It proved to give precise measurements also with low oxygen consumption rates (∼7 nmol L^−1^ h^−1^), and to significantly decrease the time required for incubations (≤14 hours) compared to traditional methods. This method provides continuous real time measurements, allowing for a number of diverse possibilities, such as modeling the rate of oxygen decrease to obtain kinetic parameters. Our data revealed apparent half-saturation concentrations (K_m_ values) one order of magnitude lower than previously reported for marine bacteria, varying between 66 and 234 nmol L^−1^ O_2_. K_m_ values vary between different microbial planktonic communities, but our data show that it is possible to measure reliable respiration rates at concentrations ∼0.5–1 µmol L^−1^ O_2_ that are comparable to the ones measured at full air saturation.

## Introduction

Oxygen is a critical parameter for life, and the respiratory oxygen consumption of living organisms has been extensively studied as a function of multiple variables. Oxygen respiration is, however, not only interesting in a physiological context, but also as a major factor in the biogeochemical cycling of carbon and in the organic matter flow through any ecosystem. Although the majority of our planet is covered by seawater, comparatively little has been done on the direct measurements of planktonic community respiration (CR). Due to the poor resolution and high detection limits (∼1 µmol L^−1^) of the applied methods for determination of O_2_ concentration (e.g. Winkler titrations, or Clark-type sensors), direct measurements of oxygen consumption rates by laboratory incubations of oceanic waters have been hard to obtain [1]. Direct respiration measurements have thus been largely restricted to the most active 200 m of the upper ocean, resulting in a database which is highly biased with respect to season, latitude and depth [2], [3]. Direct measurements of the low rates found in mesopelagic and bathypelagic oceanic waters, oligotrophic regions, and natural low oxygen waters, such the ones found in oxygen minimum zones (OMZs), are essentially missing, and we often rely on estimated values [4]–[6]. The scarcity of measurements from the twilight and dark zones of the ocean (below 150 m), which account for 98% of ocean volume and play a key role in mineralization, storage and burial of excess organic matter from the photic layer [7], have affected our ability to obtain reliable estimates of overall oceanic planktonic respiration, as well as the derived global carbon budgets [8]. An unresolved discussion still continues on the basis of controversial results, obtained from unproductive aquatic systems [9] equaling 80% of the oceans' surface. Respiration rates in such areas were estimated to greatly exceed primary production, indicating local net heterotrophy [10]–[12]. On the other hand, through a different type of data analysis, the organic carbon budget of open oceans was found to be essentially in balance regarding organic carbon [13].

Similarly, the lack of detailed data on aerobic respiration processes in oxygen minimum zone waters limits our understanding and ability to predict their establishment and development [14]. Further, in order to assess CR rates through direct measurements of oxygen consumption long incubation times (≥24 h) have often been applied, as this has been unavoidable due to the low respiration rates and the low sensitivity of the techniques. However, long incubation times may cause changes in the community structure and dynamics, leading to unrealistic estimation of rates [15], [16]. There is thus a clear neeed for more reliable and accurate mesurements, and for new methods to routinely assess CR rates from low activity waters.

Recently, a lower detection limit for respiration rates has been reported in connection with determination of microplankton respiration rates [17] by the use of a modified version of the electron transport system (ETS) enzymatic assay [18]. Over a relatively short time (3 to 5.5 h in oligotrophic waters) the activity of the ETS is estimated *in vivo* by the reduction of 2-para(iodo-phenyl)-3(nitrophenyl)-5(phenyl)tetrazolium chloride (INT). The analytical detection limit of this new method is 64 nmol L^−1^ insoluble formazan crystals (INT-F). This value is equivalent to 80 nmol L^−1^ O_2_, using a ratio between respiration rate and enzymatic activities measured by electron transport system (R/ETS ratio) of 12.8, for the conversion of ETS activity into carbon respiration. This method should therefore overpass the limits of the standard ETS assay, by measuring the true in situ physiological rate rather than the *in vitro* potential respiration. Despite its improved detection limit, the validity and accuracy of this type of enzymatic approaches for respiration measurements have been severely questioned [6].

During the recent years, new sensors for high precision measurement of oxygen concentrations have been developed. High resolution STOX sensors and high sensitivity optical sensors (optodes) are thus suitable and promising tools for the study of aquatic respiration [19] [20].

To overcome the technical and methodological problems associated with the previous respiration measurements in low activity and low oxygen waters, we developed and validated a procedure to directly quantify CR at low rates. This was done using high resolution STOX sensors [19] at artificially reduced O_2_ concentrations and assuming that the rates at low and high O_2_ concentration are comparable. Low O_2_ concentrations are required to achieve the highest sensitivity of the method. However, the above mentioned comparison could not be tested for low-activity oceanic waters, where the kinetics of oxygen uptake at high oxygen concentration cannot be reliably resolved with any of the standard methods. Therefore, we first carried out a comparative study in high-activity coastal waters, where oxygen consumption rates were determined both by the STOX method and by standard Winkler titrations. Thereafter, we used the STOX method to assess CR rates in oceanic water from the eastern tropical north Pacific (ETNP) OMZ. Additionally we present the kinetics of oxygen uptake from fully aerobic conditions down to almost zero oxygen from various planktonic communities; exploring a new, poorly described, range of concentrations where aerobic respiration is still occurring.

## Materials and Methods

### Ethics statement

To access and sample the water from the three Danish locations, used in this study, no special permission was required. To navigate, sample and work in Mexican waters during the eastern tropical north Pacific (ETNP) cruise (March-April 2012) aboard of R/V Thomas G. Thompson, permission was requested and obtained from the Mexican government. For all stations and all experiments no endangered or protected species were involved.

### Study sites and sampling

Seawater and brackish water samples were collected from three different locations in Denmark (Fig. S1). The two main locations (St. 1 and St. 2) were sampled during three seasons (June-July 2011, September 2011 and January-February 2013), the additional St. 3 only in February 2013, as described in the following. Station 1 is located in Randers fjord (56°31′12.22′′N; 10°13′48.59′′E), which is influenced by freshwater discharge by two rivers in its inner part, and a high organic load [21]. The salinity at the sampling site ranged from 7 to 9‰ during the samplings (June and September 2011 and January 2013). Station 2 is located in Kattegat near Marselisborg Marina, Aarhus, (56°08′14.32′′N; 10°12′55.19′′E). The sampling site is very close to the coastline, and the salinity ranged from 16 to 26‰ during the samplings (July, September 2011 and January 2013). Station 3, that was sampled in February 2013, is at the harbor in Hanstholm, on the Danish North Sea coast (57°07′12′′N; 08°37′12′′E) where the salinity was 35‰. Superficial seawater was collected, filtered through a 250 µm screen to remove larger fauna, and transported to the laboratory in clean polyethylene containers within 3 hours of sampling.

The method was also used as a shipboard experiment in the eastern tropical north Pacific (ETNP) oxygen minimum zone (Station 4, 16°29′93.25′′N, 109°59′00.59′′W) during the ENTP Spring Cruise (March and April 2012) aboard of R/V Thomas G. Thompson. Station 4 is located at about 700 km from the Mexican coast (Fig. S1). Seawater samples were collected with a 10 L Niskin bottle rosette at 110 m, just below the oxycline. At this depth dissolved oxygen was found to be undetectable by high resolution in situ oxygen profiling with a STOX sensor [22]. Bottle incubations were performed shortly after sampling, as follows.

### Set-up and experiments

**Figure 1:**
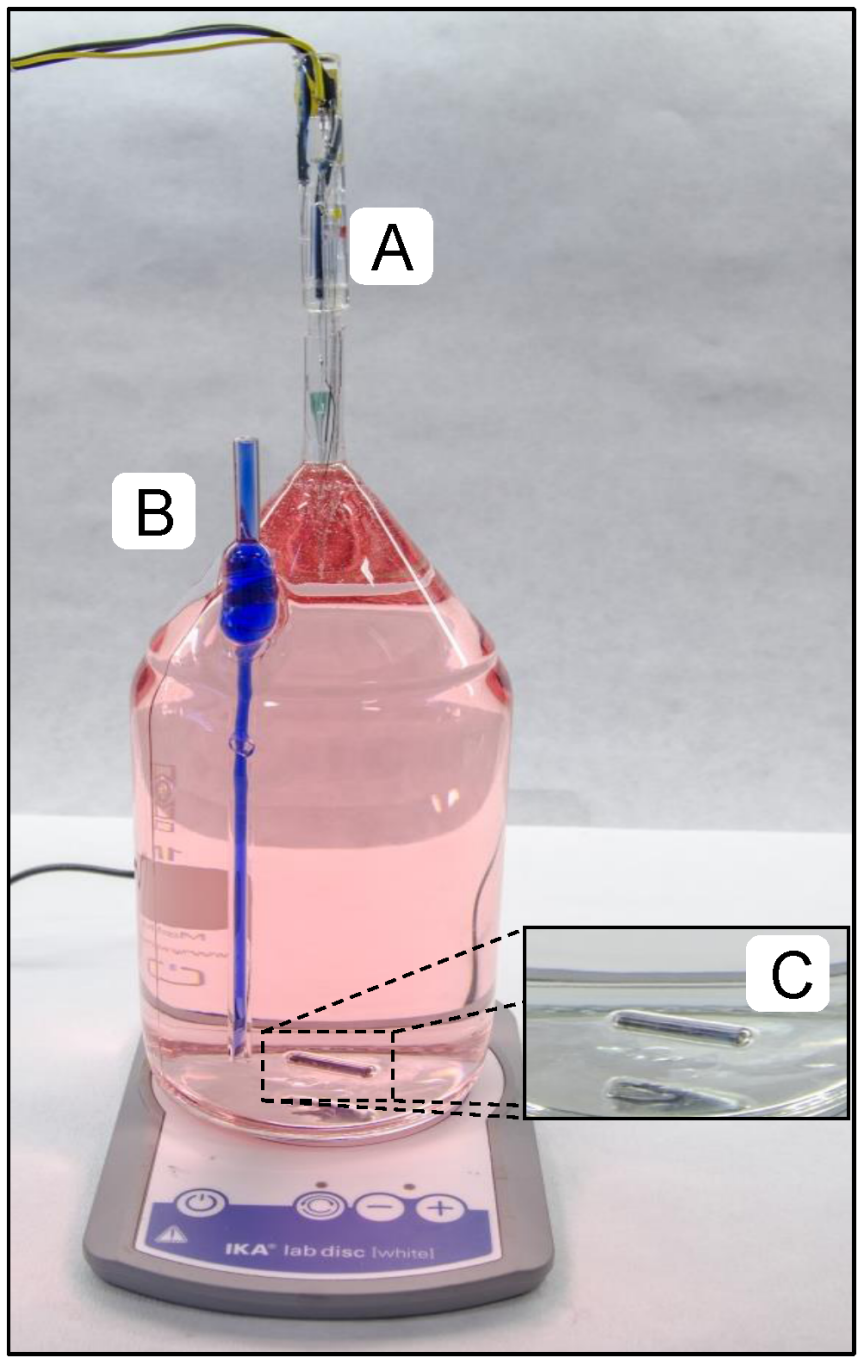
Incubation bottle. Glass bottle for incubations with a volume of 1160 mL (red ink was injected to increase the contrast). A) Opening (internal diameter 8.1 mm) for insertion of the STOX sensor; B) long open glass tube (internal diameter 2.5 mm) for pressure compensation (blue ink was injected inside the tube to increase the contrast); C) glass coated magnet (2.5 cm) for constant stirring.

Laboratory based incubation experiments were performed in custom-modified Schott Duran glass bottles of 1160 mL (Fig. 1). A 25 cm long glass tube (internal diameter 0.25 cm) penetrated the glass wall in one side of the bottle. It served for pressure compensation so that temperature volume changes did not result in bubble formation and other undesirable effects. A wider glass tube (internal diameter 0.8 cm) replaced the original neck of bottle, and it served for STOX O_2_ sensor insertion. The bottle content was in contact with air only at water-air interface at the top of the capillary (Fig. 1 B), but as the inside diameter of this capillary is small, the transport of O_2_ into the bottle is negligible (see Results section). The diameter of the STOX sensors is within 0.1 mm of the 8 mm neck of the bottles, and the contact with air was thus restricted to an ultra-thin layer of water over a distance of >3 cm (Fig. 1 A). Due to the strong capillary action between the STOX sensor and the 8-mm neck any volume changes will be visible only in the pressure compensation tube.

The bottles were kept in darkness and submersed in a water bath maintained at constant temperature (15°C and 21°C) during the experiments. Continuous stirring was applied with 2.5 cm long glass coated magnets (Fisher Scientific) (Fig. 1 C), while placing the container with the bottles on top of magnetic stirrers (IKA).

In order to obtain the highest relative resolution in the measurements of O_2_ concentration, with the STOX sensors, it is necessary to work at low O_2_ concentrations. Therefore the O_2_ concentration of the water to be investigated was lowered by bubbling with N_2_ mixed with 0.05% CO_2_.

Due to air contamination during filling, it turned out to be difficult to obtain very low O_2_ concentrations in the bottles, but the following procedure makes it possible to obtain starting concentrations down to about 100 nmol L^−1^. The water to be investigated is bubbled for about 15 minutes with N_2_ mixed with 0.05% CO_2_ while enclosed in a 10- or 20-L glass bottle with only a small opening to allow gasses to escape. The bottles are then filled from this reservoir while still bubbling, using a glass tube siphon with Tygon tubing joints. Tygon is to be preferred as it is relatively impermeable to O_2_ and transparent so that the presence of unwanted bubbles can be observed. The filling is performed through the pressure compensation tube while an inert gas stream is maintained within the bottles by a 5 mm Tygon tube inserted through the 8 mm neck. The first 50–100 mL of water flowing into the bottles are discarded by turning the bottle upside down while still maintaining the gas stream and the water inflow; afterwards the bottles are filled completely. The STOX sensor is then inserted immediately while disrupting the water flow through the siphon.

All experiments were conducted within 30 h of sampling, and incubations were kept at a maximum duration of 20 h. All the glassware was washed in first 0.1 M NaOH and subsequently in 0.1 M HCl to avoid organic contamination.

The STOX method was tested (Exp. 1) and compared with the standard Winkler technique in Danish coastal and fiord waters (Exp. 2); subsequently the STOX method was applied in oceanic waters to assess CR rates at intermediate depths of the ETNP OMZ (Exp. 3).

#### Exp. 1 - Testing the method. Control experiment for O_2_ influx and sensor stability

**Figure 2:**
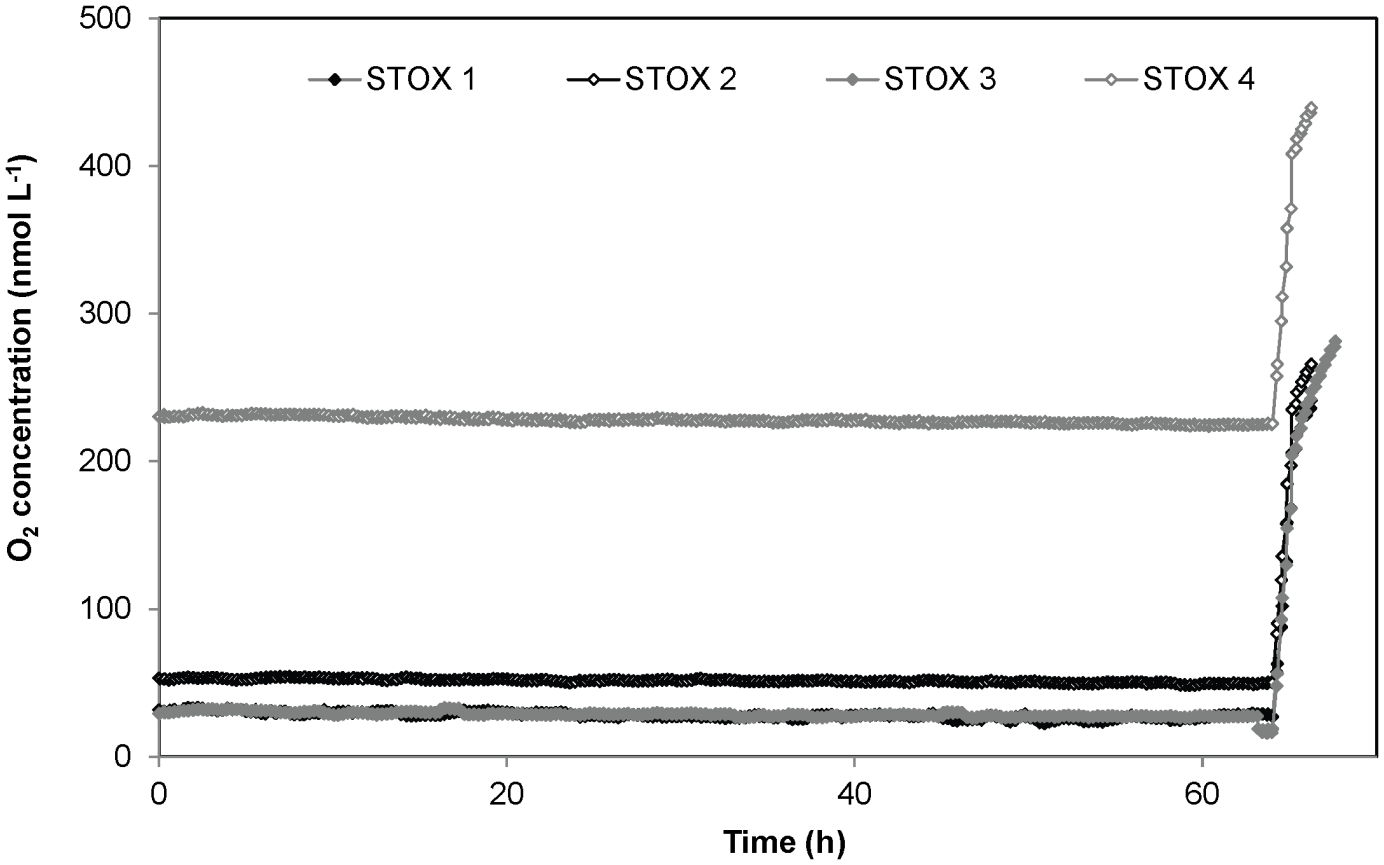
Results from the control experiment (Exp. 1). Four different replicates with different oxygen concentrations are shown. A 64 h 1 mL of air saturated 0.05 M HCl in Milli-Q water, corresponding to 244 nmol L^−1^ O_2_, was added for sensor calibration.

In Exp. 1, the above described set-up was used for evaluating the accuracy and resolution of the method, and for checking for possible sources of external and internal O_2_ contamination. A control experiment was thus established with biologically inactive water, where O_2_ concentration was monitored in 4 replicate bottles filled with 0.05 M HCl in demineralized water, which had been boiled, cooled, and then degassed to low O_2_ levels with N_2_ gas (Fig. 2).

#### Exp. 2 - Method application and comparison in coastal waters

In Exp. 2 planktonic community respiration rates were measured with the STOX method, using the same set-up and procedure, for St. 1, 2, and 3, during three different seasons. A set of parallel incubations bottles (n = 3) was set up for St. 1, 2, and 3 in order to compare the CR rates obtained with the STOX method (at low oxygen concentrations) with the respiration rates measured at air saturation by Winkler titration. In addition, kinetic parameters for the planktonic communities were modeled from the data obtained by the STOX method. For incubations at air saturation the same procedure as the one described above was repeated, but in this case the water was gently bubbled with air and the STOX sensor was replaced with a solid glass bar to close the bottle. Samples for oxygen determination via Winkler titration were taken in 12 mL Exetainers (n = 6) before and after 24 hours of incubation. The O_2_ concentration was determined spectrophotometrically following the procedure described by Labasque et al. [23].

#### Exp. 3 - Method application in oceanic waters

In Exp. 3, planktonic community respiration rates were measured with the STOX method for samples from the oceanic St. 4 in the Pacific Ocean (Fig. S1).

### Chlorophyll *a* extraction

For each station chlorophyll *a* was extracted from the sampled water before the beginning of the bottle incubations, by filtering ∼1 L of seawater through Advantec GF-75 glass fiber filters with subsequent extraction of the pigment with 96% ethanol [24]. The measurements were made in four replicates, and chlorophyll *a* concentration were calculated according to the Lorenzen method [25].

### Sensor: Principle, calibration and electronics

The STOX sensor is an amperometric oxygen sensor with built-in capability for in situ zero calibration. The sensor is composed of an internal oxygen microsensor [26] within an external microsensor casing equipped with an additional silicone membrane. Between the two membranes, an outer cathode made of porous gold (front guard) is placed. The polarization of this front guard at −0.8 V consumes all the oxygen diffusing from the external medium to the measuring sensor, resulting in an in situ zero current determination. The subsequent depolarization of the front guard allows oxygen to pass through and reach the measuring cathode. The measured difference in current is proportional to the oxygen concentration. Since a new zero reading is being recorded for each measurement cycle, the signal is independent of zero drift and allows for a definition of zero O_2_ concentration. The high accuracy in the zero determination allows for very high resolution of low O_2_ concentrations, whereas other methods are to be preferred at concentrations close to air saturation. The STOX sensors have high stirring sensitivities, and relatively long response times for a full cycle (20 s up to few minutes) compared with standard oxygen microsensors, thus they are most suited for analysis in well-stirred media with relatively slow changes in O_2_ concentration such as in the bottle incubations described here. The microsensors were built as described previously [19], [27]. Calibration was performed during each experiment, for every sensor, by injecting known volumes of air saturated water into the bottles. The sensors were connected to a PA8000 eight-channel picoammeter (Unisense A/S, Denmark), while the polarization and depolarization of the front guard were regulated by a custom-built timer-controlled switchbox with the timer set to 250 s for front guard on, 250 s off cycles. The signals were collected by a Unisense ADC816 16-bit A/D converter, connected to a portable PC using the program Sensortrace Basic (Unisense A/S, Denmark) (Fig. S2).

### Data analysis and modeling of kinetic parameters

The O_2_ concentration was continuously recorded in each set of incubations for a maximum of 20 hours. O_2_ consumption rates were calculated from linear regression of oxygen concentrations over time. Slopes were calculated by running slope over discrete O_2_ intervals for the data obtained by STOX method. For O_2_ concentrations above 6 µmol L^−1^, 2 µmol L^−1^ intervals were used, and progressively lower concentration ranges were applied with decreasing O_2_. The fit to the linear model was evaluated by the coefficient of determination, r^2^.

The kinetic parameters, V_max_ (maximum respiration rate) and K_m_ (apparent half-saturation constant) of the Michaelis-Menten equation: 
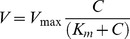
(1)were estimated by performing non-linear parametric fits directly on the progress curves (concentration versus time) for each replicate. Data were fitted to Equation (2), which describes the progressive uptake of oxygen over time [28]: 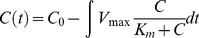
(2)


Where C(t) is the oxygen concentration as a function of time, and C_o_ is the initial concentration for the considered interval. V_max_ and K_m_ were varied iteratively until the best fit, obtained by least squares adjustment, was achieved using Solver command in Excel [29].

To test whether the kinetic parameters changed over time, K_m_ and V_max_ values were determined twice, in the same bottle and in several experiments, by injecting air saturated water when the O_2_ concentration approached zero.

In an attempt to obtain a better fit between model and experimental data we also tested an empirical relationship, originally developed for light saturation curves for photosynthesis [30]. The Jassby and Platt approach express respiration rates versus oxygen concentration as follows:
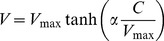
(3)


Where α is the initial slope of the curve at low oxygen levels. The equation was modified in order to use the half-saturation constant (K_m_) by the following relationship, which express the initial slope of a rate versus concentration curve obeying Michaelis-Menten kinetics:
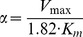
(4)


Combining equations 3 and 4:
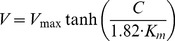
(5)


Similarly to the use of Michaelis-Menten kinetics, the time evolution of the oxygen concentration in the incubations was fitted to the equation:
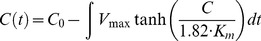
(6)


Following the same iterative procedure as described above.

## Results

### Method assessment

#### Exp. 1 - Testing the method. Control experiment for O_2_ influx and sensor stability

Oxygen consumption determinations at low O_2_ concentrations requires extreme precision, and a set-up completely free from external and internal O_2_ contamination [31]. To determine oxygen consumption at low O_2_ levels we developed an all-glass incubation bottle, with an easy access for sensors, where the only possible entry of O_2_ is through the 25 cm long pressure compensation capillary and through the thin water film between sensor and 8 mm glass tube (Fig. 1). The pressure compensation tube is 2.5 mm wide, and calculation of the oxygen flux by Fick's first law (Eq. S1, Text S1) results in an entry of 0.05 nmol L^−1^ h^−1^ at equilibrium, which is totally negligible. From the beginning of the incubation the water in the tube had the same O_2_ concentration as in the bulk water, and equilibrium will not be approached within many months if diffusion is the only means of transport [32]. The viscosity of water does, however, decrease with increasing temperature, and it may be necessary to use pressure compensation tubes with smaller internal diameter at higher temperature to minimize turbulent mixing. In addition to turbulent mixing, transport through the pressure compensation tube may also be increased by changes in temperature resulting in water movements in the tube. Water flow in a tube is slower along the walls, and any flow will thus promote mixing. The other entry point is the water film between STOX sensor and surrounding glass tube. Assuming a water film thickness of 0.05 mm, in the >3 cm long tube, only 0.02 nmol L^−1^ O_2_ h^−1^ will at equilibrium pass this film by diffusion. However, as with the pressure compensation tube we cannot exclude the possibility for turbulent mixing, for example caused by vibrational pumping. As we thus cannot exclude various types of mixing in the open connections to the atmosphere we decided to test for possible leaks into the incubation bottles by control experiments with biologically inactivated 0.05 M HCl demineralized water, which had been boiled, cooled and then degassed to low O_2_ levels with N_2_ gas. The oxygen concentration in the bottles (n = 4) did not increase for up to >60 hours (Fig. 2), which confirms that there was no oxygen leakage. On the other hand, the sensor signals decreased slightly during the incubation. Depending on the specific sensor, we registered a decrease in signal (after 64 h of incubation) ranging from 2.2% to 21.3% of the initial values (average of the signal during the first 5 h). Thus for experiments with incubations lasting 14 hours, an average drift in signal around 1.8% should be taken into account. The drift is highly dependent on the specific features of the sensors, and it could thus be possible to minimize it by using only sensors that have been thoroughly tested and kept polarized for several hours before use. Sensor drift seems, however, to be causing only part of the measured decrease in O_2_ concentration. Analyzing the data in Fig. 2 in more detail, it appears that the decrease in O_2_ in the bottles: 0.14, 0.07, 0.04 and 0.15 nmol L^−1^ h^−1^ for STOX 1, 2, 3 and 4 respectively (Fig. 2) was not strongly correlated with the initial O_2_ concentration. It thus appears that most of the decrease could be due to our inability to completely stop the O_2_ consumption, biological or chemical. Here we are working in a range of concentrations and absolute rates where control experiments are difficult, but based on low sensor signal drift rates at atmospheric concentration [19], a drift of about one third of the signal within 3 days, as observed in STOX 1 (Fig. 2) is also very unlikely. Our best estimate of the real sensor drift during the incubation is thus <2% per 14 h.

#### Exp. 2 - Method application and comparison in coastal waters

**Figure 3:**
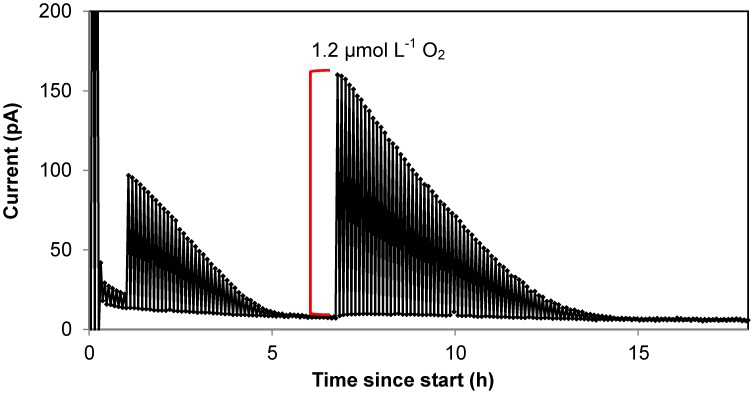
Time course of oxygen depletion in a STOX sensor incubation with coastal seawater. Example of STOX sensor data from incubation of water from Station 2 (Marselisborg Marina, September 2011). Only minimum and maximum readings during each cycle are plotted and connected with lines. The difference between maximum and minimum readings is used as a measure of the O_2_ concentration. The initial amplitude of the signal (11 pA) corresponds to an O_2_ concentration in the bottle of 200 nmol L^−1^. Injections of air saturated water were made at 1 h (2 mL, ∼600 nmol L^−1^), and at 6.7 h (4 mL, ∼1200 nmol L^−1^), as showed by the red line.

**Figure 4:**
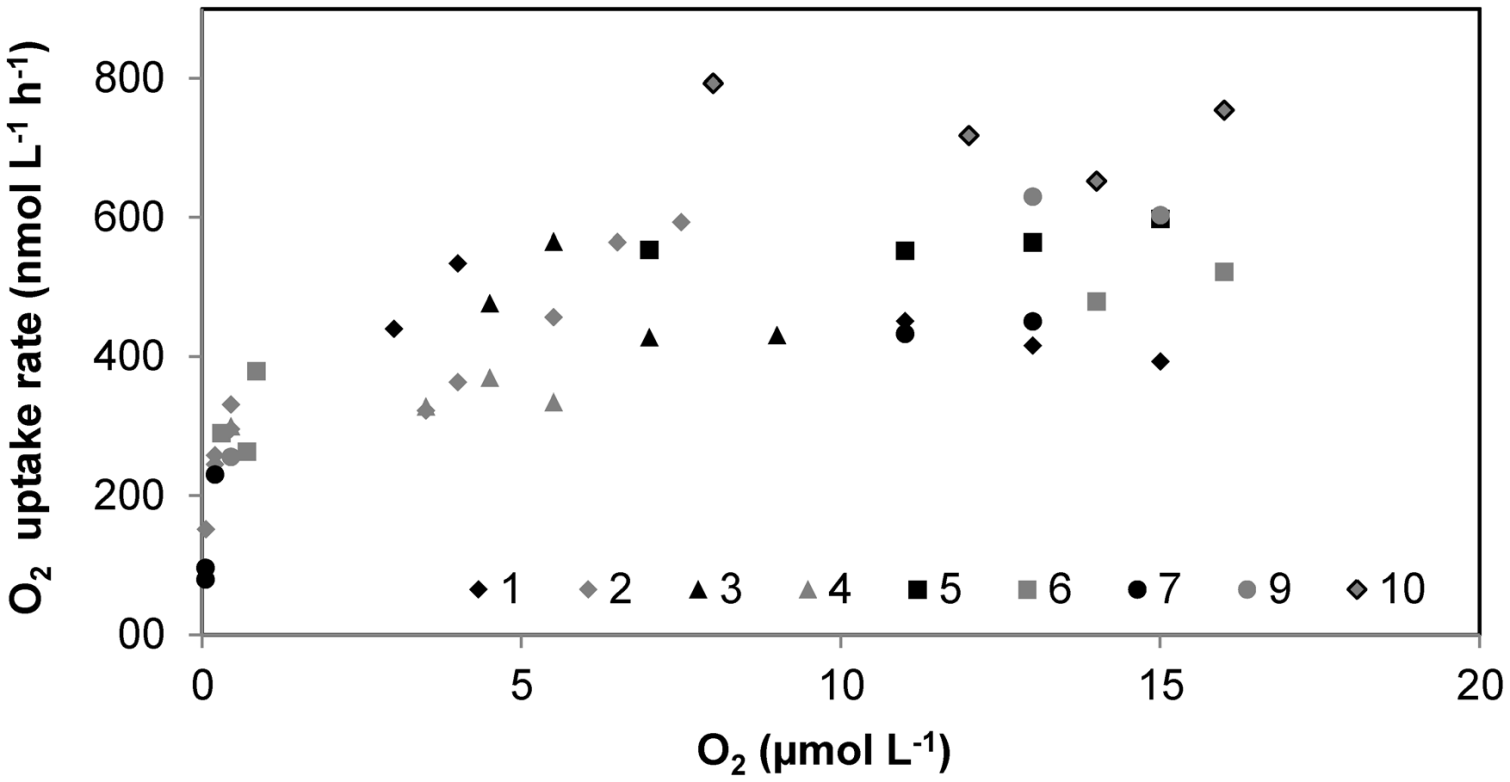
O_2_ consumption rate versus O_2_ concentration from Station 1 (Randers Fjord, September 2011). Each type of symbol corresponds to a specific bottle. Replicate bottles were incubated over range of initial O_2_ concentrations varying from 0 to 20 µmol L^−1^. Rates were calculated as slopes over different O_2_ concentration change intervals: from 17 - 6 µmol O_2_ for every 2 µmol L^−1^ intervals, from 6 - 1 µmol L^−1^ O_2_ for every 1 µmol L^−1^ intervals. Afterwards over the following intervals: 500 - 300, 300 - 100, and 100 - 5 nmol L^−1^.

**Figure 5:**
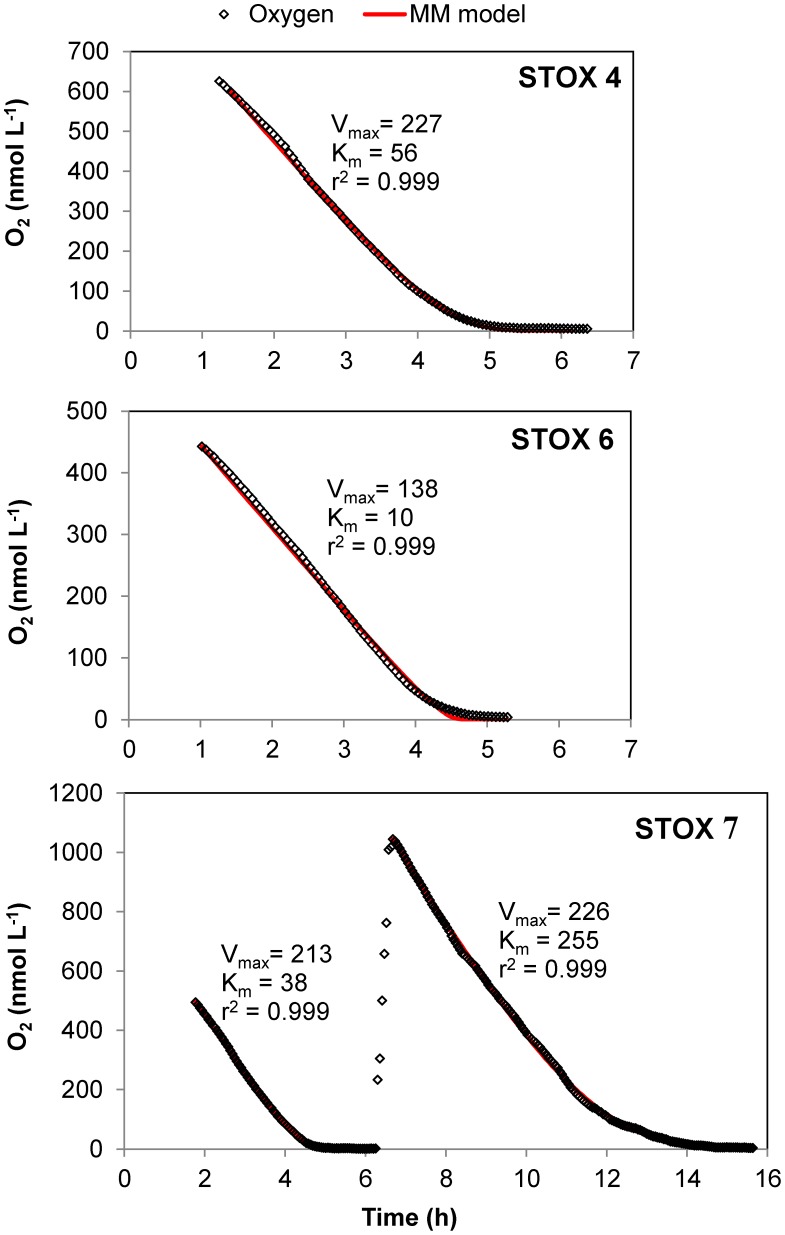
Examples of modeling of oxygen depletion curves in coastal seawater samples. Oxygen depletion experiments from three incubation bottles (4, 6, 7) from St. 2 (Marselisborg Marina), September 2011. Air saturated water was injected in bottle “STOX 7” once O_2_ was depleted (6 h). The dots show O_2_ values recorded by the STOX sensor. Maximum rates (V_max_) and half saturation constants (K_m_) were determined by least squares fits (red solid line) using Eq. (2), derived from Michaelis-Menten kinetics. The method gave a relatively poor fit that might result in an underestimation of the K_m_ value for the “STOX 6”.

The method was experimentally tested by measuring CR rates for 3 different stations in Danish coastal waters and over 3 different seasons, in order to cover a minimum variability in community composition and level of metabolisms. The main two stations of this study (St. 1, Randers Fjord and St. 2, Marselisborg Marina, Fig. S1) were sampled in June-July, September and January, and three sets of reactor experiments were performed. Since our method allows to continuously record oxygen concentration over time, we were able to monitor and analyze the oxygen consumption process with a high resolution rather than with few points (Fig. 3). It was thus possible to assess that oxygen decrease in the reactors was linear from high O_2_ concentrations down to oxygen concentrations ≤1 µmol L^−1^. Respiration rates during incubations over a wide range of O_2_ concentrations appeared to match the general shape of a rectangular hyperbola (Fig. 4), and to follow Michaelis-Menten kinetics. The parameters V_max_ and K_m_ were determined by curve fitting using Eq. 2 (Fig. 5). Estimated V_max_ values and apparent half saturation concentrations, K_m_, for St. 1 and 2 are reported in Table 1 for the three study periods.

**Table 1:** Overview of measured community respiration rates.

Sampling location	Temp. (°C)	Chl a (µg L^−1^)	V_max_	K_m_	n	r^2^
St. 1 (June 2011)	21	8.38	761±76	146±15	7	1.000±0.000
			*605±94*	*104±16*		*0.995±0.003*
St. 1 (September 2011)	21	2.28	336±41	90±14	5	0.999±0.001
			*262±38*	*66±14*		*0.999±0.000*
St. 1 (February 2013)	15	0.73	298±24	166*±*25	3	0.995*±*0.001
			*223±13*	*107±11*		*0.993±0.001*
St. 2 (July 2011)	21	2.29	309*±*49	259*±*100	6	0.998*±*0.001
			*238±26*	*155±39*		*0.998±0.001*
St. 2 (Sept. 2011) (1–6 h)	21	0.83	233*±*19	179*±*49	1	0.999*±*0.001
			*206±17*	*150±39*		*0.999±0.000*
St. 2* (Sept. 2011) (6–14 h)	21	0.83	277 *±*11	310*±*61	5	1.000*±*0.000
			*226±23*	*234±64*		*0.999±0.000*
St. 2 (January 2013)	15	0.80	186*±*21	93*±*25	3	0.997*±*0.001
			*156±15*	*84±18*		*0.997±0.001*

Summary of average V_max_ (nmol L^−1^ O_2_ h^−1^), K_m_ (nmol L^−1^ O_2_) ± standard error, number of replicates in brackets, for St. 1 and 2 in both summer and autumn. Incubation temperatures in the laboratory are reported. R^2^ values assess the fit between the data and the Michaelis-Menten and Jassby (Italic) models used to estimate the apparent V_max_ or CR rates, and K_m_ values of the communities. St. 2* refers to V_max_ and K_m_ values estimated with data recorded after injection of air saturated water, during the second progress curve in the same incubation.

**Figure 6:**
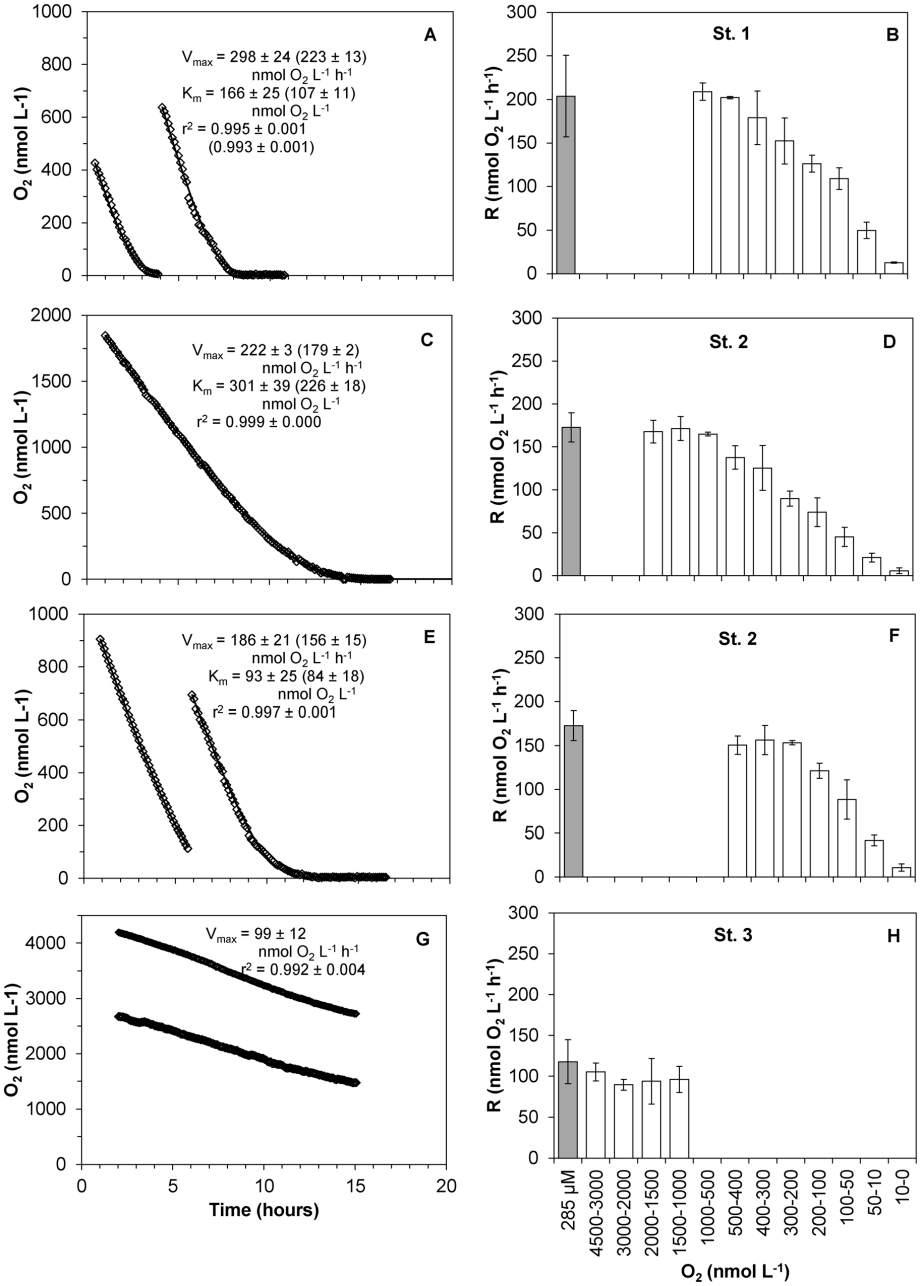
Comparison of CR rates measure with the STOX method and with Winkler titrations. Time course of oxygen depletion during incubations (A, C, E, G) and respiration rates at different O_2_ concentrations (B, D, F, H) measured by the STOX method (from 0 to 2 µmol L^−1^, white bars), and by Winkler titrations (at air saturation, grey bars). A, B: St. 1 (Randers Fjord); C, D: St. 2 (Marselisborg Marina) at concentrations of 0–2 µmol L^−1^ and E, F: Marselisborg Marina at concentrations of 0–1 µmol L^−1^. G, H: St. 3 (Hanstholm). Rates are averages ± standard error (n = 3). Both Michaelis-Menten kinetic parameters and Jassby and Platt ones (in parenthesis) are presented.

The respiration rates measured at reduced O_2_ concentrations by the STOX sensor method and at air saturation by Winkler titrations led to similar results. In fact, the two methods recorded about the same O_2_ uptake rates, as long as the O_2_ concentration in the bottle incubations with the STOX sensors was above 500 - 200 nmol L^−1^, depending on the different communities (Fig. 6). Below these values respiration rates were dependent on the O_2_ concentration, indicating that the communities were approaching their apparent K_m_ values. This methods comparison was carried out for St. 1 and St. 2, and the additional St. 3 in the North Sea, with similar results, during winter 2013 (Fig. 6). Samples from St. 1, St. 2 and St. 3, exposed to O_2_ concentrations between 2 – 0.5 µmol L^−1^ resulted in similar oxygen uptake rate (within standard errors) as the ones exposed to 285 µmol L^−1^ O_2_. Measurements with the STOX sensors and Winkler titrations, respectively, thus yielded the following respiration rates ± standard error (n): for St. 1, 196.8±9 (3) nmol L^−1^ h^−1^ and 203.8±27 (3) nmol L^−1^ h^−1^; for St. 2, 163.5±2 (13) nmol L^−1^ h^−1^ and 172.7±8.5 (4) nmol L^−1^ h^−1^; for St. 3, 98.6±4.9 (6) nmol L^−1^ h^−1^ and 117.7±13.5 (4) nmol L^−1^ h^−1^. For the planktonic community of St. 2, incubations carried out in lower O_2_ (≤500 nmol L^−1^) seemed to reach the maximum rates at slightly lower concentrations than the ones exposed to higher O_2_ levels (≤2 µmol L^−1^) (Fig. 6 D and F).

Apparent K_m_ values determined after oxygen depletion and subsequent injection of air saturated water were similar to those obtained for the first O_2_ depletion curves for St. 1 in June and September. In detail, for St. 1 in June, the average apparent K_m_ value ± st. error (n) for the first depletion curve was 83±11 (3) nmol L^−1^ O_2_, and after the addition of approximately 1 µmol L^−1^ O_2_ and its subsequent consumption, the apparent K_m_ value was 118±25 (3) nmol L^−1^ O_2_. In September, the apparent K_m_ value was first 73±25 (3), then 85±26 (3) nmol L^−1^ O_2_, after the injection of approximately 0.5 µmol L^−1^ O_2_. For St. 2 in September, however, the second oxygen depletion curve resulted in apparent K_m_ values that increased from 179 in the first run to 310 nmol L^−1^ O_2_ in the second run (Table 1, and Fig. 5 “STOX 7”).

Both St. 1 and St. 2 exhibited pronounced reductions in the respiration rates only when the concentration of oxygen in the bottles reached the nanomolar range, corresponding to apparent K_m_ values for the O_2_ respiring microbial communities between 259 - 90 nmol L^−1^ O_2_. The estimated V_max_ values for the two locations were different during summer/autumn/winter (Table 1) and varied between 186 and 761 nmol L^−1^ h^−1^. Similar values for K_m_ and V_max_ were obtained by modeling the data according to the mathematical function (Eq. 6) derived from the Jassby and Platt empirical approach, but the kinetic parameters were, in general, somewhat lower and resulted in a better description of the experimental data (Fig. S3). Using this approach, the estimated V_max_ values correspond better to the actual measured respiration rates (CR) than the values obtained using the Michaelis-Menten model. The two locations showed the same seasonal pattern for CR: summer/autumn/winter: 605/262/223 nmol h^−1^ (St. 1), and 238/206/156 nmol L^−1^ h^−1^ (St. 2), and K_m_ values varied between 155 - 66 nmol L^−1^ O_2_. The chlorophyll *a* concentrations (Chl *a* – Table 1) showed a change in abundance of the phytoplanktonic community over the seasons, and also between St. 1 and St. 2. The chl *a* concentration was by far the highest in the June 2011 sample from Randers Fjord (St. 1), which also exhibited a far higher respiration rate than the other samples (Table 1). A linear correlation was found between chl *a* concentration and CR for St. 1 (r^2^ = 0.99).

#### Exp. 3 - Method application in oceanic waters

**Figure 7:**
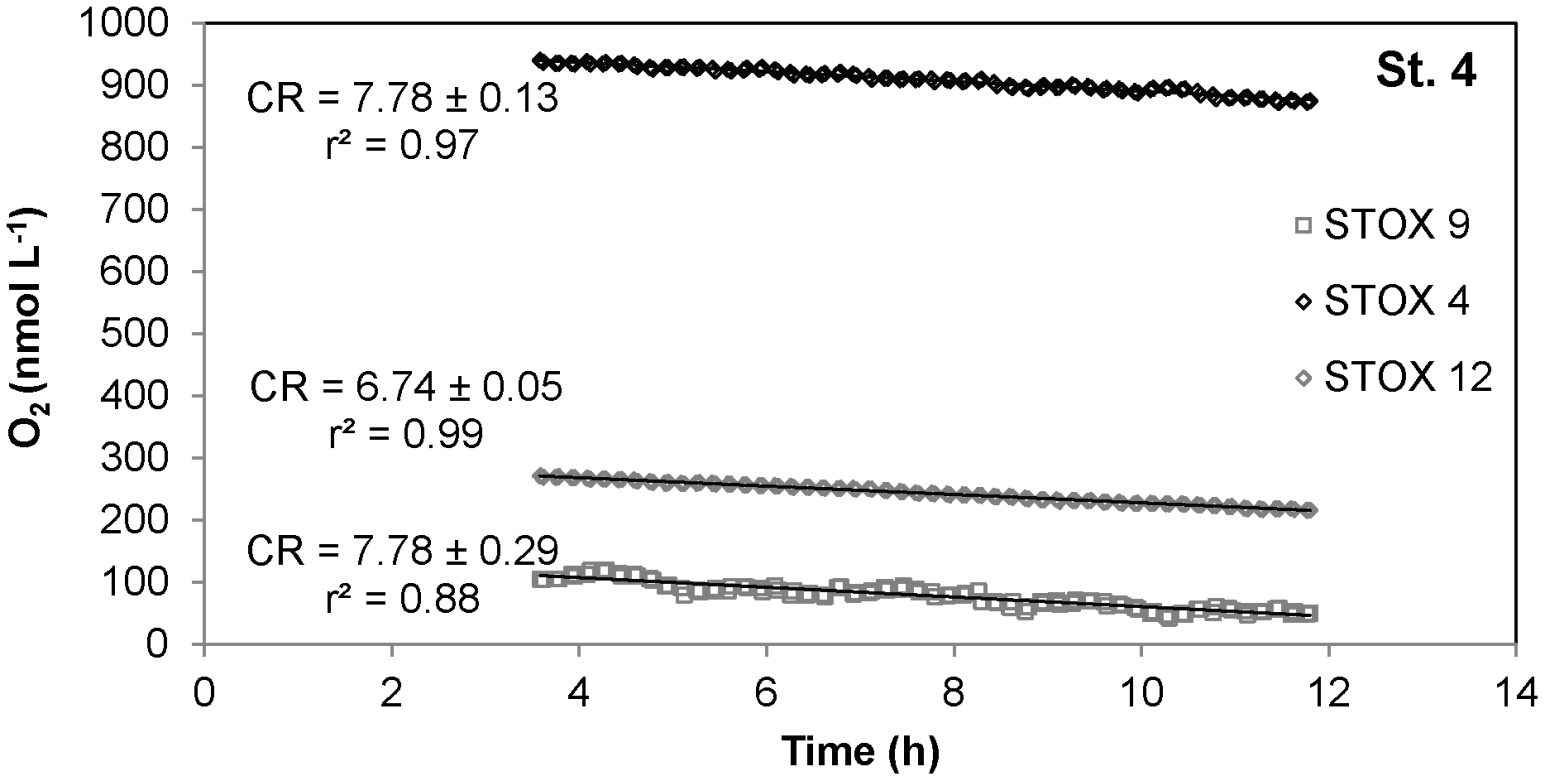
Time course of oxygen depletion in a STOX sensor incubation with oceanic seawater. Time course of oxygen depletion, in 3 replicate bottles, during shipboard experiments from oceanic Station 4, April 2012. The 3 samples, incubated at 3 different levels of DO, expressed similar CR rate (nmol L^−1^ h^−1^). CR rates ± standard error were determined by linear regression, the first three hours of the experiment being discarded due to noise on the sensor signal.

The method was tested on oceanic water samples by measuring CR rates in a shipboard experiment. Water samples were incubated in different bottles at various oxygen levels (Fig. 7). A linear oxygen decrease was detected in the three replicates bottles over 14 hour incubation time. The respiration rates were similar in the replicates, with an average value of 7.4±0.3 (3) nmol L^−1^ h^−1^, independently of the O_2_ levels (950/270/100 nmol L^−1^) at which it was exposed. This would suggest that this community has apparent K_m_ values below 100 nmol L^−1^.

## Discussion

In this study we present a new method for the determination of aerobic respiration rates in planktonic communities, applying a combination of ultrasensitive O_2_ determination, in this case by the STOX sensor, and bottle incubations with artificially lowered O_2_ concentration. By this method the resolution of O_2_ consumption rates is inversely proportional to the O_2_ concentrations within the optimal working range of the STOX sensors, i.e., from saturation down to about 10 nmol L^−1^ O_2_. A detection limit in respiration rate of about 0.4 nmol L^−1^ h^−1^ can be obtained at 100 nmol L^−1^ O_2_, assuming that the drift in signal over a 14 h interval is less than 2% (Fig. 2) and a resolution of about 2 nmol L^−1^ h^−1^ may be obtained at 500 nmol L^−1^ O_2_. This should be compared with Winkler titrations, where the accuracy in the difference between initial value and final value at best is about 0.15 µmol L^−1^ [2]. For a 14 h incubation, this corresponds to a detection limit of about 10 nmol L^−1^ h^−1^. Expansion of the incubation time lowers the detection limit by the Winkler method much more than it does for the STOX-based method presented here, but we have seen several-fold increases in respiration rates if incubation times exceed 20 h [22]. The STOX-based method thus increases the sensitivity of O_2_ respiration measurements by more than an order of magnitude as compared to the previous direct measurements. It thus allows also for determinations of the extremely low rates of deep oceanic waters, oligotrophic regions and hypoxic zones, down to about 1 nmol L^−1^ h^−1^ [33]. CR rates below 10 nmol L^−1^ h^−1^ were measured over ≤14 h long bottle incubations for the planktonic community inhabiting intermediate depths of the ETNP OMZ off the coast of Mexico (Fig. 7).

A good agreement was found between the rates measured with this method and the ones obtained from traditional Winkler titrations (Fig. 6), confirming the validity of this new approach. Further, this agreement indicates a lack of a detectable immediate effect on CR rates by artificially lowering the oxygen concentration in the samples over a short time interval; as long as the samples are exposed to DO considerably above the apparent K_m_ values of the communities. In addition this method, with its high resolution and continuous measurements of oxygen consumption, allows for a unique possibility to study kinetic parameters in natural samples, and the response of planktonic communities to varying O_2_ levels.

The respiration rates measured for the three stations in Denmark (Fig. S1) (0.16–0.8 µmol L^−1^ O_2_ h^−1^) are in the same range as earlier estimates or measures for the upper mixed layer of the open ocean [1]. Surface water (<10 m) in the investigated coastal areas was found to respire at an average rate of 0.3 µmol L^−1^ O_2_ h^−1^, equivalent to the previous average value from a combined dataset for coastal-ocean areas (0.3 µmol L^−1^ O_2_ h^−1^) [2], [16], [34]. The stations showed different levels of activity as it could be expected from the locations, which are subjected to different degrees of eutrophication and organic loading. St. 1, which exhibited the highest uptake rates, is located in a long shallow estuary, influenced by freshwater discharged by two rivers with high organic load [21], and likely harbors a very active planktonic community. St. 2, which exhibited lower respiration rates, is located just outside Aarhus harbor, in coastal Kattegat seawater, with a lower organic load and less dense planktonic community; and St. 3 is located in the relatively less active waters of the North Sea. Lastly, St. 4 is an example of the low rates found in open oceanic waters. The still relatively high oxygen consumption at St. 4 can be attributed to the presence of a deep secondary chlorophyll maximum at the sampled depth (110 m) co-occurring with a higher density of cells (see Ulloa et al. 2012 [35] for general description of the secondary chlorophyll maximum). Furthermore, the differences in respiration rates also reflect the seasonality of the planktonic activity, higher in summer months and lower in autumn and winter, following a similar trend as the one registered for the seasonal fluctuation in chl *a* concentration (Table 1).

The purpose of this investigation was, however, not to determine accurate rates of respiration in Danish coastal waters, but to investigate the validity of the approach for respiration rate determinations using water with an artificially reduced O_2_ concentration, and to determine the kinetics of O_2_ uptake in marine and estuarine water at low O_2_ concentrations. Therefore we did not try to closely simulate the in situ temperature conditions, nor to investigate the temporal change in respiration rate as a function of the diurnal light cycle where respiration is generally higher just after the light period [36]. We decided to estimate the kinetic parameters for O_2_ uptake by directly modeling the O_2_ concentration over time in order to avoid the inaccuracy and limitations of linearization processes [37], as well as the consecutive errors of using dependent variables and inferred values (i.e. slopes), instead of measured ones. The Michaelis-Menten model seemed to give a fair description of the kinetics of the investigated mixed planktonic communities, although the equation does not strictly apply to heterogeneous populations with different kinetic parameters [38].

By applying Michaelis-Menten kinetics, we assume that diffusion limitation in aggregates can be neglected. In fact, when bacteria aggregate they experience lower external O_2_ concentration, due to the consumption of the neighboring organisms. In the extreme case of a thick biofilm the O_2_ uptake can be described by half-order kinetics [39]. There will always be some aggregation in planktonic communities, and this will result in apparent K_m_ values that are higher than the cellular ones. The diffusion to a cell becomes less efficient with increasing cell size [40], and we can therefore expect higher apparent K_m_ values for large cells. A major proportion (>70%) of the community respiration in seawater can, however, be attributed to organisms from size class ≤10 µm, with up to 58% from the size class smaller than 1 µm, usually dominated by bacteria [41]–[44]. Due to the not perfectly met assumptions of the Michaelis-Menten model, the curves modeled using Eq. 2 give very good fits to the experimental data, but result in a mismatch between the modeled V_max_ and the experimentally detected maximum respiration value. We therefore tried to apply the empirical model of Jassby and Platt (1976), and in this case better agreement was found. The main difference between the two models is that by the modified Jassy and Platt model the maximum rate (V_max_) is approached at lower concentrations for the same assumed K_m_ values (Fig. S3). The original model was developed to better simulate the behavior of mixed microbial communities such as those investigated in this study.

The apparent K_m_ values estimated during our reactor experiments are lower than the ones previously reported for marine bacteria [45]. Nevertheless, half saturation constants for O_2_ in this order of magnitude are a wide spread phenomenon in the microbial world [46]–[49], as well as the ability to obtain energy from aerobic respiration at extremely low O_2_ concentrations through high affinity terminal oxidases [49]–[51]. In fact, the reduction of O_2_ is such an exergonic reaction that it can provide enough free energy to fuel the cellular processes also at extremely low O_2_ concentrations [50]. It is thus not surprising that microorganisms may use oxygen as electron acceptor as long as it is available, even at concentrations of few nanomolar [52].

Metagenome analysis of marine samples, from both open ocean and a coastal area, indicated a very low abundance of high-affinity terminal oxidases having K_m_ values of a few nanomolar, whereas analysis of cultured bacteria indicated presence of such oxidases in 93% of the 734 investigated aerobes [49]. The absence of such high-affinity terminal oxidases in marine waters is not surprising, as most of the ocean is fully oxic and only bacteria in the guts of fauna [53], in large fecal pellets and marine aggregates [54]–[56] are exposed to highly reduced ambient O_2_ concentrations, where they may experience O_2_ concentrations in the nanomolar range. On the other hand, high-affinity terminal oxidases have been recently reported in oxygen minimum zones waters by Kalvelage et. al. [57]. Low affinity terminal oxidases have, however, K_m_ values around 200 nmol L^−1^, so even planktonic communities without any high-affinity terminal oxidases can be expected to have such relatively low K_m_ values. Coastal environments with a more diverse bacterial community and oceanic oxygen minimum zones with a low-O_2_ adapted community can be expected to be characterized by apparent K_m_ values somewhat below the 200 nmol L^−1^ as also indicated by some of our data.

There may be some dependence of the respiration rates on the pretreatment of the water. This was seen from the diverging estimates of the K_m_ values in a second oxygen depletion experiment (Fig. 5 and Table 1: St. 2*, Sept. 2011, 6–14 h). Prolonged exposure to complete anoxia is one factor that should be avoided, as it resulted in often very different rates and K_m_ values. Also, the data from the first 1–2 h of incubation should be discarded as the community should be allowed to adapt to the new conditions [58]. It can be recommended to use data from between 2 and 16 h for determination of kinetic parameters. Incubations exceeding 16–20 h may result in highly erroneous data, at least with open ocean water samples (L. Tiano et al. in prep.) [22].

We are aware of the limitations of in vitro measurement of microbial processes [15], [16], [59], but the use of the STOX sensor in the presented set-up provides an excellent solution to several of the problematic issues associated with previous determinations of seawater respiration rates. In fact, it is a simple and accurate way to assess planktonic respiration in real-time, free from the use of conversion factors and estimated values. The Electron Transport System (ETS) method, for example, is such an indirect method where measured potential respiration rates must be converted to *in vivo* respiration rates by empirically determined algorithms [60]. Another indirect approach is estimations from particulate organic matter (POM) fluxes [33], which are subject to many biases [5], [61].

The high sensitivity of the STOX sensor it allows for very short incubation times. In addition, the decrease of O_2_ in the reactors was linear at concentrations above about three times the estimated K_m_ values, throughout the duration of the experiments, which implies a steady-state community [16]. Thus we excluded major changes in the community composition often associated with long term incubations [62].

Electrochemical sensors have been applied in this study, but it is also possible to use new generation optodes. These newly developed very sensitive optodes represent a valuable alternative to the STOX sensors [20]. Irrespective of whether electrochemical sensors or optodes are used, careful in situ calibration should be performed, both before and after the recording of O_2_ consumption to verify the absence of sensor fouling. In fact, aggregates may form around tips of STOX sensors; this phenomenon was observed in diatom-rich coastal water (L. Tiano, personal observation) and biofilms may form on optode surfaces, leading to erroneous measurements.

By use of the technique described in this paper it is possible to compare rates of O_2_ uptake with rates of other microbial metabolic processes such as rates of N-transformations obtained by high resolution ^15^N isotope techniques, and to study the transition from aerobic to anaerobic respirations [63], [64]. We can now also in detail investigate how defined low O_2_ concentrations affect gene expression in natural environments such as seawater (T. Dalsgaard, personal communication) or in pure cultures [22].

## Supporting Information

Figure S1 **Map of sampling sites.** The STOX incubation method was tested with samples from 3 stations in Danish coastal and fiord waters (left side map). Danish sampling locations (•): Station 1, Randers fjord, Station 2, Marselisborg Marina, Station 3, Hanstholm. All samples were taken within a 100 m distance from the coast/shore line. The method was also used to assess planktonic community respiration rates in water samples from the eastern tropical north Pacific (ETNP) oxygen minimum zone (right side map). Station 4, located ∼700 km from the Mexican coast. The map shows isoclines representing oxygen concentrations expressed in % of saturation at 150 m depth, oxygen data were derived from World Ocean Atlas 2009, National Oceanographic Data Center (USA) (Ocean Data View).
(TIFF)

Figure S2 **Schematic drawing of incubation set-up.** Set-up for monitoring oxygen consumption, including: 1) Shift box. 2) Power supply for the front guard. 3) ACD816bit A/D converter (Unisense A/S), 4) PA8000 Multi-channel picoammeter (Unisense A/S), 5) Computer, 6) Water bath placed over magnetic stirrers (IKA, lab disc) containing glass bottles with STOX sensors and glass coated magnets. It is possible to fit all the equipment necessary for an experiment with up to 8 replicates bottle and 8 STOX sensors, on about a meter of lab bench space.
(TIFF)

Figure S3 **Comparison of kinetics models.** Upper plot: respiration rate vs. oxygen concentration, a comparison of Michaelis-Menten and Jassby and Platt equations using the same fixed parameters. Lower plot: time evolution of the oxygen concentration in the incubation of one sample from Station 2, and comparison of Michaelis-Menten and Jassby and Platt equations, using as V_max_ the respiration rate measured at 100% oxygen saturation (173 nmol L^−1^ h^−1^).
(TIFF)

Text S1 **Oxygen consumption of STOX sensor.**
(DOC)

Text S2 **Comparison and assessment of the use of different kinetic models.**
(DOC)
