# Supplementary figures and images for: A New Highly Sensitive Method to Assess Respiration Rates and Kinetics of Natural Planktonic Communities by Use of the Switchable Trace Oxygen Sensor and Reduced Oxygen Concentrations
Source: PLoS One. 2014 Aug 15;9(8):e105399. doi: 10.1371/journal.pone.0105399 (PMC4134296; doi:10.1371/journal.pone.0105399)

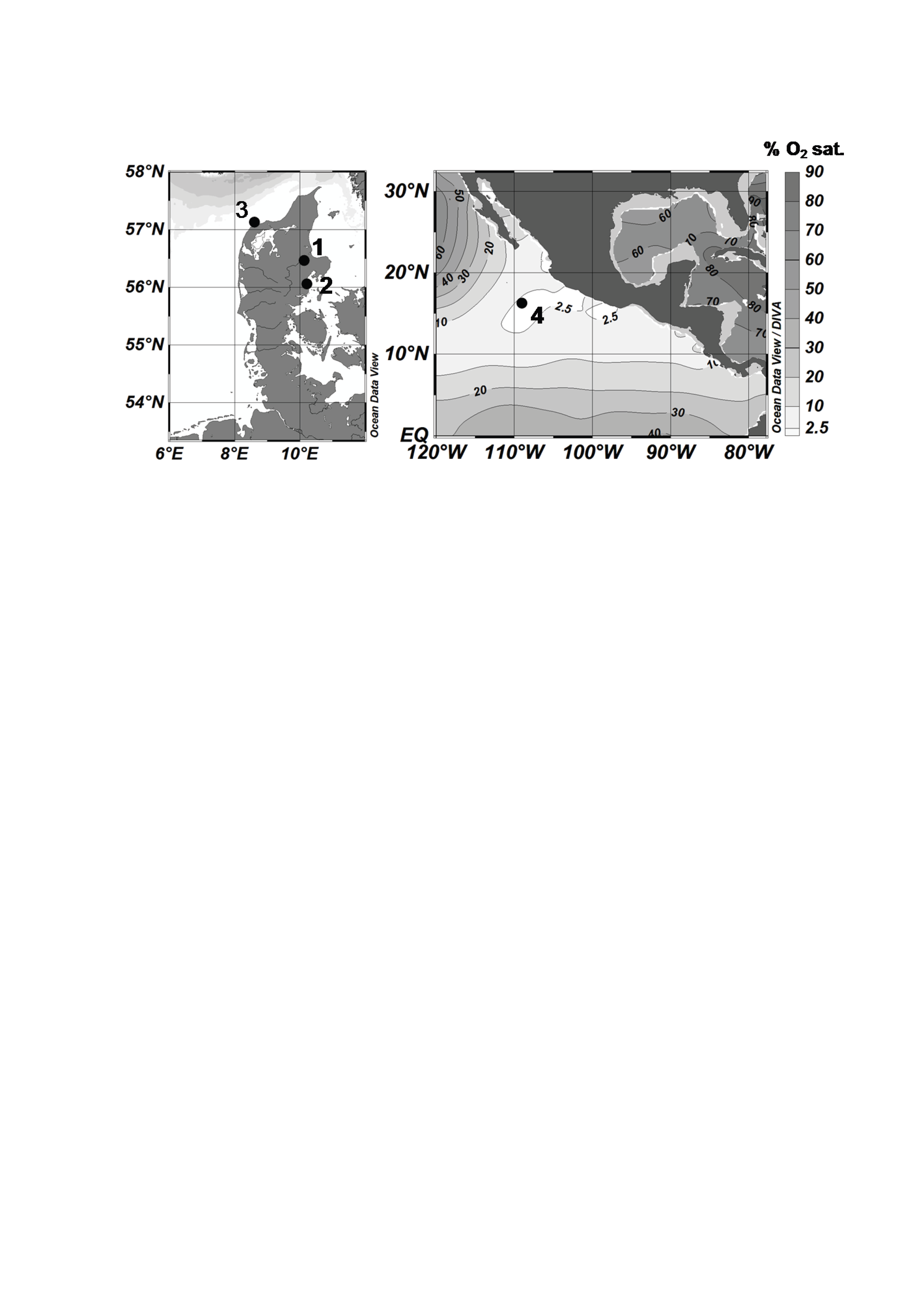

Supplement: Figure S1 — Map of sampling sites. The STOX incubation method was tested with samples from 3 stations in Danish coastal and fiord waters (left side map). Danish sampling locations (•): Station 1, Randers fjord, Station 2, Marselisborg Marina, Station 3, Hanstholm. All samples were taken within a 100 m distance from the coast/shore line. The method was also used to assess planktonic community respiration rates in water samples from the eastern tropical north Pacific (ETNP) oxygen minimum zone (right side map). Station 4, located ∼700 km from the Mexican coast. The map shows isoclines representing oxygen concentrations expressed in % of saturation at 150 m depth, oxygen data were derived from World Ocean Atlas 2009, National Oceanographic Data Center (USA) (Ocean Data View). (TIFF) [file pone.0105399.s001.tiff]

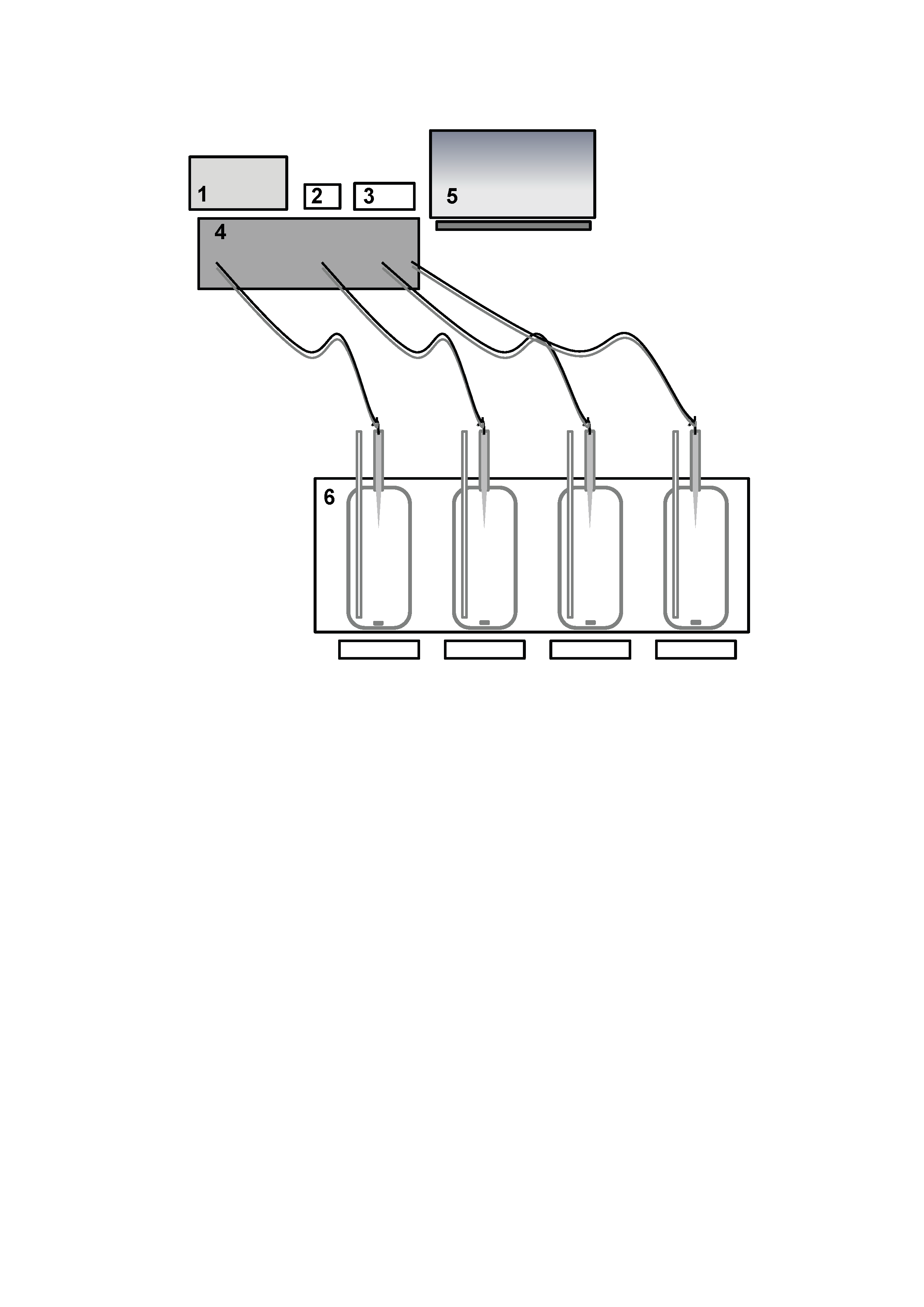

Supplement: Figure S2 — Schematic drawing of incubation set-up. Set-up for monitoring oxygen consumption, including: 1) Shift box. 2) Power supply for the front guard. 3) ACD816bit A/D converter (Unisense A/S), 4) PA8000 Multi-channel picoammeter (Unisense A/S), 5) Computer, 6) Water bath placed over magnetic stirrers (IKA, lab disc) containing glass bottles with STOX sensors and glass coated magnets. It is possible to fit all the equipment necessary for an experiment with up to 8 replicates bottle and 8 STOX sensors, on about a meter of lab bench space. (TIFF) [file pone.0105399.s002.tiff]
