# Supplementary material for: A New Highly Sensitive Method to Assess Respiration Rates and Kinetics of Natural Planktonic Communities by Use of the Switchable Trace Oxygen Sensor and Reduced Oxygen Concentrations
Source: PLoS One. 2014 Aug 15;9(8):e105399. doi: 10.1371/journal.pone.0105399 (PMC4134296; doi:10.1371/journal.pone.0105399)
Supplement: Text S1 — Oxygen consumption of STOX sensor. (DOC) [file pone.0105399.s004.doc]

**Supporting text S1.**

**Oxygen consumption of STOX sensor.** The maximum oxygen consumption of the STOX sensor is produced when the front guard is polarized and all the oxygen coming through the front membrane is consumed right behind it. Therefore, the maximum sensor consumption can be calculated as the oxygen flux diffusing through the silicon membrane to a complete anoxic internal environment by applying Fick’s first law of diffusion:

$J\left(O_{2}\right)=-D\frac{\partial c}{\partial z}$ (S1)

Considering a large STOX sensor with a tip membrane diameter of 100 µm and a membrane thickness of 20 µm, with the silicone having a 5 timers higher permeability than water (equivalent to a diffusion coefficient of 10^-4^ cm s^-1^ at the same O_2_ concentration as in water ), it will have an oxygen consumption of 4 nmol h^-1^ when inserted into air saturated (285 µmol L^-1^) water. This value represents 0.001% of the total O_2_ content of the modified bottle used in this study (1160 mL). Since the oxygen consumption is always proportional to the external O_2_ concentration, i.e. about 0.001% of the total pool, this consumption can be neglected with incubation times of less than 24 h.

**REFERENCES**

1. Zhang H, Cloud A (2006) The permeability characteristics of silicone rubber. In Proceedings of the 2006 SAMPE Fall Technical Conference "Global Advances in Materials and Process Engineering", Coatings and Sealants Section. Dallas, TX: Society for the Advancement of Material and Process Engineering.
