# Supplementary material for: A New Highly Sensitive Method to Assess Respiration Rates and Kinetics of Natural Planktonic Communities by Use of the Switchable Trace Oxygen Sensor and Reduced Oxygen Concentrations
Source: PLoS One. 2014 Aug 15;9(8):e105399. doi: 10.1371/journal.pone.0105399 (PMC4134296; doi:10.1371/journal.pone.0105399)
Supplement: Text S2 — Comparison and assessment of the use of different kinetic models. (DOC) [file pone.0105399.s005.doc]

**Supporting text S2.**

**Comparison and assessment of the use of different kinetic models.** The shape of the O_2_ consumption rates versus O_2_ consumption curves during the incubations appears to be similar to the rectangular hyperbolic curve expected for enzymatic reactions. Due to such hyperbolic curves, a Michaelis-Menten relationship has been usually assumed for microbial respiration processes . However, Longmuir showed more than half a century ago that respiration rates approached a maximum at a lower concentration than predicted, and that the Michaelis-Menten relationship should ideally only be applied from 10 to 90% of the maximal respiration rate .

A significant effort has been made in order to find various mathematical expressions resulting in a better curve fit. Jassby and Platt found that the fit of Michaelis-Menten equation to light saturation curves for photosynthesis resulted in a poor description of the experimental data and they proposed an alternative mathematical expression based on a hyperbolic tangent function. We tested this latter expression with our data, modifying the original equation in order to use a K_m_ value, instead of the photosynthetic efficiency (α), used in the original expression.

A comparison of a traditional Michaelis-Menten model and the one derived from the Jassby and Platt equation, using the same parameters: V_max_ = 100 and K_m_ = 100, is shown in the upper part of Fig. S3. In both cases, the K_m_ value represents the concentration which results in half of the maximum rate, but clear differences can be observed. Simulations obtained by the Jassby and Platt equation approach the maximum rate within 1% at 4 - 5 times the K_m_ value whereas this happens for much higher concentrations by the Michelis Menten expression (Fig. S3). Using the respiration rates at 100% air saturation from one of the incubations of Station 2, the oxygen concentration as a function of time was modeled, applying both equations (Fig. S3). V_max_ was fixed (173 nmol L^-1^ h^-1^) and the K_m_ value was iteratively calculated as described in the Methods section to get the best fitting by the least squares method. The use of Jassby and Platt equation resulted in a perfect fit to the real data (r^2^ = 0.999), whereas the Michaelis-Menten equation resulted in a visible deviation (Fig. S3). However, the Michaelis-Menten model would also have a good fit, if V_max_ is changed from 173 to 216 nmol L^-1^ h^-1^ (r^2^ = 0.999), but this results in a mismatch between the modeled V_max_ and the experimentally detected maximum respiration value.

In the majority of our replicate bottle incubations, both Michaelis-Menten and Jassby and Platt models resulted in very good fit to the experimental data (similar r^2^ values). The kinetic parameters (both V_max_ and K_m_) estimated by Jassby and Platt model were, however, always lower than the one obtained with the Michaelis-Menten model.

Our data suggest that when dealing with O_2_ uptake kinetics of mixed communities the modified Jassby and Platt equation might yield better approximations to experimental data as compared to the Michaelis-Menten model. Future experiments will show whether this is universally true.

**REFERENCES**

1. Devol AH (1978) Bacterial oxygen uptake kinetics as related to biological processes in oxygen deficient zones of the oceans. Deep Sea Research 25: 137-146.

2. Longmuir IS (1954) Respiration Rate of Bacteria as a Function of Oxygen Concentration. Biochem J 57: 81-87.

3. Longmuir IS (1957) Respiration Rate of Rat-Liver Cells at Low Oxygen Concentrations. Biochem J 65: 378-382.

4. Petersen LC, Nicholls P, Degn H (1974) The effect of energization on the apparent Michaelis–Menten constant for oxygen in mitochondrial respiration. Biochemical Journal 142: 247-252.

5. Jassby A, Platt T (1976) Mathematical formulation of the relationship between photosynthesis and light for phytoplankton. Limnology and Oceanography 21: 540-547.
